# How users of indwelling urinary catheters talk about sex and sexuality: a qualitative study

**DOI:** 10.3399/bjgp14X680149

**Published:** 2014-05-27

**Authors:** Alison Chapple, Suman Prinjha, Helen Salisbury

**Affiliations:** Nuffield Department of Primary Care Health Sciences, University of Oxford, Oxford.; Nuffield Department of Primary Care Health Sciences, University of Oxford, Oxford.; Nuffield Department of Primary Care Health Sciences, University of Oxford, Oxford.

**Keywords:** catheters, sex, sexuality, body image, general practice, primary health care

## Abstract

**Background:**

An indwelling urinary catheter can solve the problem of incontinence and may be life-saving in individuals with retention, but it can cause problems such as infection and may have a negative impact on body image, sex, and sexuality.

**Aim:**

To explore the individual’s perceptions of how a long-term urinary catheter can affect body image, sex, and sexuality; and to help GPs to discuss the subject in consultations.

**Design and setting:**

Qualitative study of a diverse sample of individuals living with a long-term urinary catheter. Interviews took place all over the UK, usually in the individuals’ homes.

**Method:**

Narrative interviews were audiorecorded, transcribed, and analysed thematically, using the constant comparative method.

**Results:**

Some individuals said that sex was not an important part of their lives because of old age, illness, or the catheter. Others talked about how their catheter and their disability affected their sexual self-esteem, feelings of masculinity or femininity, and how the catheter caused pain, discomfort, or unexpected symptoms during sex. Many noted the lack of information on the subject and also said that health professionals were reluctant to talk about sex. For a minority a catheter was not a major problem in relation to sex.

**Conclusion:**

Some individuals using a urinary catheter would benefit from information on how to have a sexual relationship with a catheter in place and a chance to discuss the subject with their doctors. GPs need to be aware that sex may matter to a person with a catheter and how illness, disability, and a catheter may affect sexuality.

## INTRODUCTION

Incontinence is ‘*matter out of place*’,^1^ a taboo subject in our society, a condition typically associated with old age and disability, often stigmatising both patient and carer.^2^ Treatment with a long-term indwelling catheter may enable someone to leave the house without embarrassment and it can be life-saving in individuals with retention. However, a catheter may cause physical problems such as infection,^3^,^4^ and may also impair body image and sexuality.^5^

Conditions and associated treatments that have made a catheter necessary can themselves affect sex and sexuality.^6^ For example, in multiple sclerosis neurological changes can affect sexual response. Fatigue and physical disability may compound these problems.^7^ In men with prostate cancer, surgery or hormone treatment may affect their libido, erections, and sense of masculinity.^8^,^9^ However, a good sexual experience may not require penetrative sex.^10^ Body image may be affected by the way society defines masculinity or femininity, although a person with disability may redefine masculinity or femininity in their own terms.^11^ However, GPs may shy away from discussing sex with individuals with disabilities such as a spinal cord injury (many of whom will have a catheter), either because they do not think it is pertinent, or because they lack knowledge about sexual dysfunctions, or because they fear that mentioning sexual issues may offend their patients and even jeopardise the doctor–patient relationship.^12^–^14^

Most research concerning long-term catheterisation has focused on complication rates in terms of urinary tract infections (UTIs), the development of cancer, the risk of injury to the bowel, and catheter malfunction such as blockage.^4^,^15^ Numerous studies have explored the subjective perceptions of individuals using a long-term urethral or suprapubic urinary catheter,^16^–^22^ but relatively few have focused specifically on self-image, sex, and sexuality. Recent European Guidelines for best practice in urological health care note ‘a lack of research on how sexual intercourse is affected by catheter use’.^23^ It seems that there is also a lack of research on how catheter use may affect other aspects of sexuality, such as orgasm. This article aims to explore individuals’ perceptions of how a long-term urinary catheter can affect those aspects of an individual’s life, and to help GPs to raise the subject in their consultations.

## METHOD

### Recruitment of interview participants

Individuals who had lived in the UK with an indwelling catheter for ≥3 months were invited to take part in a study of individuals’ experiences of ‘living with a urinary catheter’. Potential participants were recruited through urologists, specialist nurses, expert advisory panel members, support organisations, and ‘snowball sampling’ through personal contacts. Those who were interested received an information sheet, introductory letter, reply slip, and envelope. All participants gave informed consent before taking part and agreed to publication of their interview data.

**How this fits in**

There is scant research on how catheter use may affect sex, sexuality, and body image. This qualitative study found that sex still matters to many individuals living with a catheter, but that they lacked information and found doctors reluctant to discuss the subject. GPs could help by raising the subject or by referring patients to relevant counsellors or websites.

### The sample

The aim was to include a diverse sample of individuals from different backgrounds and areas (Table 1). Thirty-six individuals were interviewed by experienced qualitative researchers with a social science background. Patients used a catheter for various reasons (Table 2); 28 had experienced a urethral catheter at one time or another, 26 a suprapubic catheter, and 18 had experienced both.

**Table 1.** Sociodemographic characteristics of the sample

	**Sex**	

	**Male, *n***	**Female, *n***
**Age at interview, years**		
22–40	5	5
41–60	5	5
61–80	10	3
81–96	2	1

**Occupation (or previous occupation if retired)**		
Professional/managerial	10	7
Other non-manual	4	4
Skilled manual	4	0
Other: unemployed, volunteers, students	4	3

**Ethnic group**		
White British	21	14
South Asian	1	0

**Table 2.** Reason for using a catheter

**Reason**	**Individuals, *n***
Spinal cord injury	14
Neurological condition (such as multiple sclerosis)	8
Prostate problem (such as cancer)	6
Fowler’s syndrome/retention of urine	4
Endometriosis in the bladder	1
Spinal muscular atrophy	1
Bowel cancer (surgery affected bladder)	1
Uterine cancer (radiotherapy affected bladder)	1

### Interviews

Interviews took place in England, Wales, and Scotland during 2011–2013. Having signed a consent form individuals were asked to talk for as long as they liked about their experiences of living with a catheter; they were asked to ‘tell their story’. They talked about a range of issues, including their feelings about having a catheter. At this stage a few individuals talked about the way the catheter affected sex and intimate relationships.

After this narrative part of the interview, an interview guide was used to explore relevant issues that had not already been discussed. The guide reminded the interviewer to try to bring up the subject of social life, body image, and sex. For example, one question on the guide was, ‘How does a catheter affect your social life?’ However, the interviewers raised the subject of sex in a number of ways. For example, when talking to a young woman with endometriosis the interviewer said: ‘*You don’t have to talk about it if you don’t want to, but has sex been possible with a catheter?*’ The same interviewer broached the subject with a young woman with Fowler’s syndrome. She said: ‘*What about boyfriends? Does that become difficult?* ’ Sometimes questions were vague, but led to a response about sexual relationships. The interviewer used this approach when talking to a middle-aged man who had a spinal cord injury. She said: ‘*Can you tell me a little bit about going out on your own, socialising and the considerations that are important for you?* ’ His reply included an explanation of how a catheter could affect sexual relationships.

In some instances the interviewer did not attempt to raise the subject of sex. Edward was a 96-year-old widower who had an enlarged prostate. He had had a catheter for 7 years, ever since his stroke. In these circumstances it is understandable that the interviewer decided that a direct question about sex would not have been appropriate. However, the interviewers always ended the interview with a question that invited individuals to add anything else they thought was important about living with a catheter. The interviews lasted between 1–2 hours, were audiotaped and fully transcribed for analysis.

### Analysis

A qualitative interpretive approach was taken,^24^ combining thematic analysis with constant comparison.^25^ NUD*IST®, QSR N6 (a qualitative data-indexing package) was used to facilitate the analysis. Codes and/or categories for analysis were developed. These were initially identified from the literature and from the first few interviews. Then the rest of the transcripts were coded. As the analysis progressed, additional codes were added. Relevant QSR N6 reports were read and the coding and interpretation of results was discussed. Then relevant literature was used to develop interpretation. To protect the anonymity of individuals, pseudonyms were used in reporting the results.

## RESULTS

During the interviews about half of the responders talked about sex, intimate relationships, body image, or feelings about masculinity or femininity. The analysis revealed four main themes relevant to the subject:

- the relative importance of sex as part of everyday life;
- self-esteem and body image;
- physical reasons why the catheter affected sex; and
- lack of information.

### The relative importance of sex as part of everyday life

Sex was clearly very important for some individuals who were interviewed; particularly the young and middle aged. Rachel, aged 51, who had multiple sclerosis, said that:

‘*Health professionals should realise that for some clients sex is really important, and having a catheter can really affect it* .’

Others said that sex was not important in their lives, partly due to old age and partly due to illness or disability. Arthur, for example, who had spastic tetraparesis and a urethral catheter, explained why sex was not a problem that concerned him:

‘ *... about 85% of men who’ve reached their 70th birthday are relatively impotent, and when you’ve got a neurological complaint as well* [um] *... I haven’t lost my libido completely, but it isn’t an important part of my life. And my relationship on an emotional side is perfectly content. I have a wonderful wife who looks after me.*’

(Arthur, aged 77 years)

### Self-esteem and body image

Sexual self-esteem is likely to be tied up with how individuals define masculinity and femininity. These results found little evidence that individuals had redefined these concepts to include a person with a catheter. On the contrary Owen felt that having a catheter ‘*wasn’t manly*’ and Molly said that there was ‘*nothing feminine*’ about connecting a bag to a catheter at night. Several individuals made comments suggesting that they had a negative body image and lacked sexual self-esteem.^26^ This was partly due to their disability and partly due to having a catheter. For example, Rosie (aged 55 years) who had a spinal cord injury, said that her injury was ‘desexualising’ and that the catheter made the situation worse:

Interviewer (I): ‘*You touched on self-image. How does having a catheter and a bag affect your self-image, on top of all the other problems you might have?*’

Rosie: ‘*Well it’s pretty rubbish really. You know, I mean having a spinal injury is very desexualising. You, you stop really feeling like a woman and when you have a tube coming out of your belly* [um] *it’s hard. It’s very hard. It’s, it’s been a long process, it’s been nearly 3 years for me now but it’s still ongoing, that process of learning that I will never be the person that I used to be, the woman that I used to be, that I’ve got my tubing externally rather than internally now.*’

Rachel had multiple sclerosis. Her increasing spasticity, lack of muscle tone, changing body shape, getting older, plus her catheter seemed to have affected her sexual self-esteem:

‘*I would say that it wasn’t just the catheter that affected my body image. It was the combination of my body’s slightly changing shape because of my disability, being in a wheelchair, my spasticity, my posture, everything and losing muscle tightness in my body, so having a bit more of a flabby tummy. And that all combined with the catheter really affected my body image and made me question, “What is attractive, am I still attractive?” (...), I’d lost confidence in my own body and myself as a sexual woman.*’

(Rachel, aged 51 years)

Emily, aged 40 years, became paralysed after a skydiving accident when she was 31-years-old. For her the hardest part of being paralysed was dealing with the incontinence. She had a suprapubic catheter and could not imagine having a sexual relationship with a catheter in place because it affected her self-image. Nine years later she still felt that way about herself.

Hannah (aged 39 years) had +endometriosis. She had had an indwelling urethral catheter for 4 months. She found having a catheter ‘*dehumanising*’. With a catheter she lacked the confidence to have sex:

I: ‘*You said having a catheter is a bit dehumanising. Can you explain why?*’

Hannah: ‘*Yes. I, I think because you lose confidence almost in your body having this plastic thing hanging down that’s very unattractive really.* [Um] *I didn’t want to date or anything while I had this catheter in for you know several months, and it just doesn’t make you feel normal I think. Yeah I did really lose confidence for that time.*’

Sam broke his neck when he fell into a river. He became tetraplegic. His comments during the interview also suggest that his catheter affected self-image:

‘*Having an indwelling catheter, a suprapubic, it’s not something that you shout from the roof tops. It does have inevitably a mental consequence on sexual relationships, because you’ve got this pipe sticking out of you unexpectedly. And a bag of urine at the end of it, which is not the most romantic aspect of oneself.*’

(Sam, aged 61 years)

However, some individuals appeared to have a positive sexual self-image in spite of the catheter. Rebecca had Fowler’s syndrome, a condition that causes urinary retention in women. She equated having a suprapubic catheter with wearing a wig:

‘*I mean I’ve always said if they* [boys] *can’t handle it then they’re not worth being with, because that’s me and it’s part of me, and that’s it. You know, just like someone might wear a wig, I have a catheter. Someone, you know, might paint their toenails bright blue all the time. I have a catheter.*’

(Rebecca, aged 26 years)

Chloe (aged 22 years) also had Fowler’s syndrome. She had a suprapubic catheter for many years, but said that it hadn’t affected her self-esteem:

I: ‘*So boys were happy to take you out and there were no problems?*’

Chloe: ‘*Yes.*’

I: ‘*That’s good.*’

Chloe: ‘*Yes, it was fine.* [um] *Yes, I never really had a problem, but maybe that’s because I felt confident, because I’m a confident person. So maybe that, that’s why it didn’t matter. But maybe if I had been more reserved and worried about it, then it might have affected friends and boyfriends and things in that way.*’

I: ‘*So self-image wasn’t affected?*’

Chloe: ‘*No, I’m not like that.*’

### Physical reasons why having a catheter affected sex

It is possible to have sex with a urethral catheter in place. A man can leave a large loop of catheter at the end of the penis, so that when he gets an erection, there’s a length of catheter to accommodate the penis. The catheter can be held in place using a condom or surgical tape.

Luke had a urethral catheter at first. He taped it back along his penis when he wanted to have sex but he found the experience made him sore:

‘*Speaking as a male, with a urethral catheter you can have sex. But the way they describe to do it sounds very sore, and it is sore to do that because you have to try and tape it [the catheter] back, which is pulling a lot on it.*’

(Luke, aged 35 years)

Luke decided to have a suprapubic catheter, making sex much easier. Ruby (aged 60 years) also had a urethral catheter. She pointed out that sex did not have to include sexual intercourse and suggested that individuals talk to their doctor about it:

Ruby: ‘*Yes it has affected my sex life.* [Um] *negatively yes, which basically is all my fault and I sometimes feel very mean about it.*’

I: ‘*Can you have sex with a catheter in?*’

Ruby: ‘*Yes you can, or at least I’m told you can (...) They tell you to pin it* [the catheter] *up on you but if somebody’s moving around it’s always going to move around and* [um] *cause pain. So not overly happy with that. There are other ways of having sex, and I would advise people to talk to their doctor.*’

Sex is easier with a suprapubic catheter than with a urethral catheter because the catheter is positioned away from the genitalia. However, Rachel was anxious at first because her suprapubic catheter had been positioned lower down than she had expected, and she feared that it would get in the way during sex. She worried that she might not be able to have an orgasm. When she spoke to her surgeon he said that he had assumed that a woman would want to have it (the catheter) below the bikini line for sunbathing. Eventually Rachel’s surgeon agreed to reposition the catheter. A detailed description of what happened can be found elsewhere.^27^ Rachel was delighted that in spite of having a catheter she could still enjoy sex. She said she could still have an orgasm and ‘*that was fantastic*’. Chloe on the other hand, who had a suprapubic catheter for several years, sometimes found sex extremely painful because she had adhesions in her bladder and abdomen. Sex also led to more urinary infections:

I: ‘*Having the suprapubic catheter made sex a bit painful?*’

Chloe: ‘[um] *Not a bit. A lot.*’

I: ‘*Oh, really?*’

Chloe: ‘*A lot, a lot, yes. Like, me and my boyfriend, we used to have* [um] *conversations about it, because he used to* [um] *obviously want to do it* [sex] *more than what I wanted to do. But then he used to understand that I couldn’t be going through the pain. And also when I used to have sex as well, it used to make me more likely to have a urine infection. I used to think, “Oh, I can’t be dealing with having another urine infection. So I just won’t do anything at all.” That’s what it was like.*’

Rachel and Chloe both found that they had blood in their urine after sex; they were alarmed by this. Rachel asked doctors about it for 6 years.

‘*Each time I would have sex I would bleed into my* [um] *catheter bag. So the first time that happened I completely freaked out, we were abroad, I thought, “Oh my God what if something serious has happened.” Luckily my husband, very calm, he said, “Look just drink lots of water and let’s see what it’s like in the morning,” and it was fine, it was absolutely fine.* [Um] *and I have in the intervening years asked my consultant and my urologist, my continence nurse, and several other health professionals in that area why I bleed sometimes when I have penetrative sex and what I can do about it. And every single one up to 2 years ago, so that was* [um] *6 years of people saying, “I’ve no idea, I’ve never heard of that happening with anybody else.” And I can guarantee I will not be the only one that this has happened to but I think the problem is nobody ever discusses sex with their health professionals. So people could have been having this, women having this problem but just not wanting to talk about it with anybody.’*

(Rachel, aged 51 years)

Eventually a urologist told Rachel that the bleeding was probably due to the catheter rubbing against the bladder wall. The doctor suggested that she should close the catheter valve 5 minutes before she had sex so that she could leave some urine in the bladder which would reduce the friction of the catheter on the bladder wall. Rachel found that after following this advice she only bled occasionally.

As mentioned above, the physical position of the catheter during sex may be a nuisance even with a suprapubic catheter. When Chloe had sex she dealt with the situation using humour:

‘*So I used to* [um] *just move my tube over here till it would all be over to one side. But obviously in the heat of the moment you don’t really want to be like, “Oh, hang on, I’ve just got to move my wee bag out the way” kind of thing. But that is what it used to be like. And maybe it’s because I’m the type of person that can have a joke and stuff. I used to be like, “Oh, I’ve just got to move my wee bag out the way.” But obviously I wish I didn’t have to say that. But that is what I used to have to say. And we used to kind of like joke it off and stuff.*’

(Chloe, aged 22 years)

Thus humour was used to overcome any potential shame, embarrassment, or humiliation. When Godfrey^20^ interviewed older individuals about their experiences of having a urinary catheter she found that they also used humour to preserve their self-esteem.

### Lack of information

Many individuals, like Rachel above, complained that they lacked information, and some said that health professionals seemed reluctant to discuss sex or sexuality. Sam, who had a suprapubic catheter, helped a group of health professionals construct a questionnaire for a research study. He was surprised that no one thought to include a question about sex:

‘*I was most interested to find that, when I was working on a panel with medical people, putting together a questionnaire to do with long-term catheterisation, nobody had considered the aspect of sex. And that it might inhibit that* [sex]*, and the input on that came entirely from myself. And that was in a variety of I think about eight different aspects of the medical field, not one of them had considered that side. And it doesn’t appear on any paperwork that one sees. Why they think because you’ve had a suprapubic catheter put in, or that any other form of catheter, that that means sex is now out of the window, I don’t know. It doesn’t have to be, but no information.*’

(Sam, aged 61 years)

Chloe has had a catheter since she was 13 years old. She was interviewed when she was aged 22, and was dismayed that her urologist had never discussed sex with her:

‘*No one talks to you about that* [sex]*. And especially, maybe when I was younger, I never ever, ever, once got spoken to about sex, ever. But maybe that’s because I was younger. And maybe, maybe because my consultant was a male* [um] *and he was the right age to be my dad, and so maybe he felt as though he, because he did say to me, “You’re like my adopted daughter.” Maybe he felt as though he couldn’t even approach the conversation of sex with me, because I had grown up with him. I’d gone from a 13-year-old and I’m now 22 and I still see him, and he’s watched me grow and maybe he ... And now he knows I’ve had a child, but he’s never once said to me, “Oh, you’ve had a child” kind of thing, because I don’t think he knew what to say about it. But* [um] *obviously he knew that I was struggling with my, with the catheter because I used to say I was struggling. But he used to just say, “Oh, I’ll send you to* [um] *the gynaecologist. They can separate your adhesions.” He didn’t want to talk about the actual issues.*’

(Chloe, aged 22 years)

Chloe emphasises that she never had an opportunity to talk about sex by repeating the words ‘ever’ three times in one sentence.

## DISCUSSION

### Summary

Some said that sex was not an important part of their lives, partly because of old age, illness, or the catheter. Others talked about how their catheter and their disability affected their sexual self-esteem, feelings of masculinity or femininity, and how the catheter caused pain, discomfort, or unexpected symptoms during sex.

Some drew attention to the lack of information on the subject. They also said that health professionals were reluctant to talk about sex, though one woman suggested that individuals with sexual problems should talk to their doctor about it. A minority said that a catheter was not a major problem in relation to sex.

### Strengths and limitations

This study has some limitations. It was a retrospective study so individuals may have forgotten exactly why relationships faltered or how professionals reacted to queries about sex. All but one of the participants were from white British backgrounds; if individuals from different cultural backgrounds had been interviewed additional problems may have been raised.

A major strength of this study was that individuals were encouraged to talk for as long as they wished about subjects that mattered to them, so issues were raised that may not have emerged had a structured questionnaire been used and it may not have been understood why some individuals had certain concerns about sex or sexuality. There was a wide age range across the sample and therefore this meant that individuals were included who had no medical problems apart from their urinary difficulties. The sample was drawn from all over the UK, allowing for inclusion of the viewpoints from individuals who were cared for by many different GPs and urologists, from different areas with varying counselling and continence services.

### Comparison with existing literature

Most other studies that have explored the individual’s perceptions of living with a urinary catheter have either been very small, were conducted before suprapubic catheters were common, or have not focused on sex and sexuality. Nevertheless they contributed usefully to the literature. Roe and Brocklehurst,^17^ for example, found that no professional had voluntarily discussed sex with any of their participants, and that patients did not know that sexual intercourse was possible with a urethral catheter in place. The current results found that individuals lacked information, and that their doctors did not mention the subject of sex. More recent studies included individuals who had a suprapubic catheter. These findings support theirs, that while a sexual relationship with a catheter in place may be difficult and may affect self-image, this is not always so. Kralik *et al* 18 found that many of their participants perceived that a suprapubic catheter would be more acceptable to others because it was not positioned near the genitalia. Some of the current study’s responders felt the same way. However, Rachel said that even with a suprapubic catheter the positioning of the catheter mattered because, if too low down, it may still be very near the genitals and so affect sex. Rachel and Chloe were also alarmed when they saw blood in their urine after sex. To the authors’ knowledge similar situations have not been reported before.

### Implications for practice

Older individuals in the UK see their GP as an appropriate source of help for sexual problems, but they rarely initiate such discussions themselves.^28^ Younger individuals with chronic illness or disabilities may also want their GPs to bring up the subject of sex, finding it hard to report sexual problems.^29^,^30^ However, health professionals do not always recognise that individuals with disabilities or those with chronic illness have sexual needs, so their patients’ sexual health is often overlooked or neglected.^27^,^31^ Clinical guidelines recognise that individuals with disabilities may need the opportunity to discuss difficulties they may have in establishing or maintaining sexual relationships,^32^ but as noted earlier, health professionals may find it hard to discuss sexual issues with their patients.^12^–^14^ GPs find it particularly difficult to discuss sex with patients of the opposite sex, patients from black and minority ethnic groups, middle-aged and older patients, and non-heterosexual patients. Lack of time during the consultation is also a key barrier to initiating discussion about sex.^14^ GPs need to be aware that sexuality matters to many individuals with disabilities and that if individuals feel good about their body and are sexually satisfied they are less likely to feel depressed.^33^

GPs may find the Sexual Respect Tool Kit helpful (http://www.sexualrespect.com/). This is a Tool Kit designed to help GPs and other professionals to feel more comfortable initiating discussions around sex. The Tool Kit suggests a number of opening lines such as ‘Some people with a condition like yours finds it gets in the way of intimacy. Are you finding that?’ The Tool Kit includes an excellent training video and links to other resources. If GPs feel they can discuss sex and sexuality a poster in the waiting room on the subject may encourage patients to raise the subject.

GPs who feel they lack time or relevant skills could refer their patients to a counsellor or to a sexual medicine unit. They could also tell their patients about useful websites where professionals offer advice to individuals with a catheter who want a sexual relationship, such as one produced by the Bladder and Bowel Foundation (www.bladderandbowelfoundation.org/), or the one produced particularly for those with a disability such as a spinal cord injury (www.facingdisability.com/expert-topics/what-do-people-do-with-a-catheter-during-sex/diane-m-rowles-ms-np). GPs could also make their patients aware of a new section of Healthtalkonline (http://healthtalkonline.org/peoples-experiences/chronic-health-issues/living-urinary-catheter/topics), a website where individuals can find others who are living with a catheter talking about their own experiences of sex and intimate relationships. Therefore, there is much that GPs can do to improve quality of life for individuals living with a urethral or a suprapubic catheter. Sex is not a subject that can be ignored.
